# *Pimpinella anisum* Essential Oil Nanoemulsion Toxicity against *Tribolium castaneum*? Shedding Light on Its Interactions with Aspartate Aminotransferase and Alanine Aminotransferase by Molecular Docking

**DOI:** 10.3390/molecules25204841

**Published:** 2020-10-20

**Authors:** Ahmed S. Hashem, Marwa M. Ramadan, Amira A. A. Abdel-Hady, Stefania Sut, Filippo Maggi, Stefano Dall’Acqua

**Affiliations:** 1 Stored Product Pests Research Department, Plant Protection Research Institute, Agricultural Research Center, Sakha, 33717 Kafr El-Sheikh, Egypt; ashashem2014@gmail.com; 2 Economic Entomology Department, Faculty of Agriculture, Mansoura University, 35516 Mansoura, Egypt; marwa010@mans.edu.eg (M.M.R.); amiraali@mans.edu.eg (A.A.A.A.-H.); 3 Department of Agronomy, Food, Natural Resources, Animals and Environment (DAFNAE), Agripolis Campus, University of Padova, 35020 Padova, Italy; Stefania_sut@hotmail.it; 4 School of Pharmacy, University of Camerino, 62032 Camerino, Italy; filippo.maggi@unicam.it; 5 Department of Pharmaceutical and Pharmacological Sciences, University of Padova, 35020 Padova, Italy

**Keywords:** insect pest control, nano/bio-insecticides, *Pimpinella anisum*, red flour beetle, molecular docking, biochemical assay

## Abstract

The insecticidal activity is the result of a series of complex interactions between toxic substances as ligands and insect’s enzymes as targets. Actually, synthetic insecticides used in pest control programs are harmful to the environment and may affect non-target organisms; thus, the use of natural products as pest control agents can be very attractive. In the present work, the toxic effect of aniseed (*Pimpinella anisum* L.) essential oil (EO) and its nanoemulsion (NE) against the red flour beetle *Tribolium castaneum*, has been evaluated. To assess the EO mode of action, the impact of sub-lethal concentrations of aniseed EO and NE was evaluated on enzymatic and macromolecular parameters of the beetles, including aspartate aminotransferase (AST), alanine aminotransferase (ALT), total protein, total lipids and glucose. Finally, a molecular docking study was conducted to predict the mode of action of the major EO and NE components namely *E*-anethole, Limonene, alpha-himalachalene, trans-Verbenol and Linalool at binding site of the enzymes AST and ALT. Herein, the binding location of the main compounds in both proteins are discussed suggesting the possible interactions between the considered enzymes and ligands. The obtained results open new horizons to understand the evolution and response of insect-plant compounds interactions and their effect predicted at the molecular levels and side effects of both animal and human.

## 1. Introduction

Modern agricultural practice and food industry are strongly influenced by the need to control pest and parasites. Especially in the production of fruits and vegetables, there is a need to use different chemical products in order to ensure productivity and quality of crops. On the other hand, despite the usefulness of pesticides, their use imposes a range of risks, including potential harmful side effects on humans, residues in food crops, environmental pollution, development of resistance and outbreak of insect pests [1,2,3]. Actually, in order to increase the food safety and reduce the environmental impact of agricultural practices, there is an urgent need to develop new pest control agents that present improved characteristics in terms of selectivity, environmentally acceptability, biodegradability and safety of use. Natural products in fact are at the forefront as safe sources for the development of pest control agents [4,5]. Among them, the usage of essential oils (EOs), which are complex mixture of small-sized, lipophilic and volatile compounds, appears to be very promising. EO of parsley family (i.e., Apiaceae or Umbelliferae) have been studied for their insecticidal properties [6,7,8,9] showing activity against a wide range of target insects, e.g., from stored grain to insect pests, owing to their documented ovicidal, larvicidal, and adulticidal toxicity [10,11,12,13]. Nevertheless, EOs-based pesticides can present some issues, as low water solubility, rapid environmental degradation, and lack of stability during storage [14,15]. These shortcomings greatly impair the potential use of these products for pest management purposes. On this basis, their chemical and physical properties can be modified and improved by a nanotechnology approach through the development of suitable formulations [16,17,18,19,20].

Aniseed (*Pimpinella anisum* L., Apiaceae) can be considered a good material for the development of plant-based formulations for insect pest management for several reasons. The species is a well-known annual herb with one of the oldest histories of human use. It is cultivated in Egypt, which is the world’s largest producers of its EO, followed by Greece, Italy, and the Middle East [21,22,23,24]. Aniseed is also widely used as a food, being eaten directly or boiled for drinking or used as a seasoning on raw or cooked foods [25]. Being edible, this product presents a good toxicological profile. The aniseed EO is popular in the folk medicine for a wide range of therapeutic uses dealing with neurologic, digestive, gynecologic and respiration disorders [26,27]. In addition, it exhibited efficacy against stored grain insects [28,29]. The chemical composition of aniseed EO is variable in its qualitative and quantitative composition, as commonly expected for other EOs [30,31]. Indeed, the main component is (*E*)-anethole at various percentages [32,33,34], besides other compounds at a lower rate, such as methyl chavicol, *p*-anisaldehyde, α-himachalene, and (*E*)-pseudoisoeugenyl 2-methylbutyrate [24,28,35].

Overall, the insecticidal activity of EOs and their components are frequently assessed against insect pest species at the macromolecular level [9]. Nonetheless, the underlying mode of action of these EOs and their main constituents is still poorly understood with scarce studies exploring some biochemical targets and their mediated biochemical changes, such as the contents of total protein, total lipids, glucose, activity of aspartate aminotransferase (AST) and alanine aminotransferase (ALT). Here, the red flour beetle, *Tribolium castaneum* Herbst (Coleoptera: Tenebrionidae), a key pest species of stored products, was employed as a model organism [36].

As an attempt to mitigate some of the shortcomings associated with the potential use of EOs as pest management tools in the present work we aimed to establish the toxicity of aniseed EO and its nanoemulsion (NE) against the red flour beetle *T. castaneum*, and to investigate possible macromolecular abnormalities induced by the sublethal exposure to EO and NE, including changes in AST and ALT activity, glucose content, total proteins and total lipids levels. Furthermore, we investigated which EO constituent may be endowed with protease activity by using 3D molecular modeling with in silico molecular docking tools to understand their binding patterns. Overall, results from this study can aid us in understanding the mode of action of aniseed EO, when encapsulated in NE, allowing a better understanding to its metabolism and distribution.

## 2. Results

### 2.1. GC-MS Analysis of P. anisum Essential Oil and Nanoemulsion

**Figure 1:**
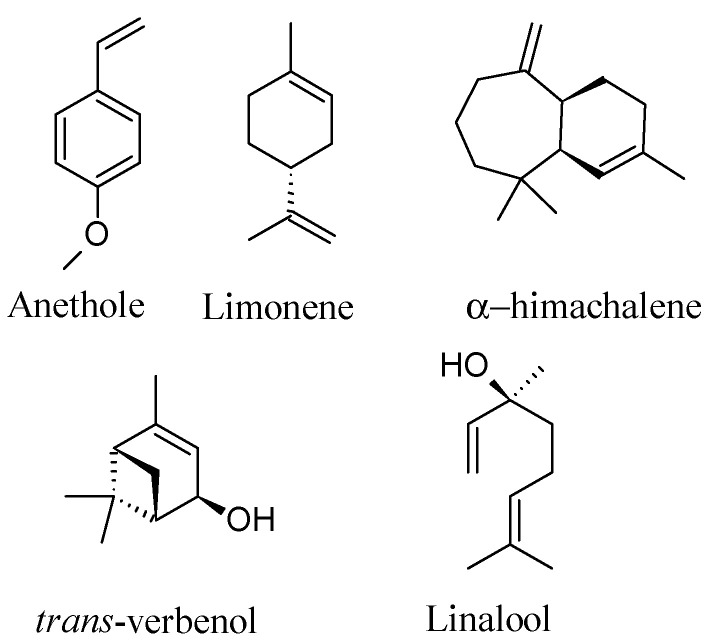
Structures of the main compound of the analyzed essential oil.

The results of the GC-MS analysis of *P. anisum* EO and NE showing the composition of both the samples is reported in Table 1, with the identification and quantification of fourteen different volatile constituents, accounting for more of the 99% of oil components. In the aniseed EO, the predominant component as expected is (*E*)-anethole accounting 800 mg/g), followed by limonene, α-himachalene, trans-verbenol, linalool, eugenol, acetyl isoeugenol and methyl chavicol. On the other hand, In the aniseed NE, the dominant component with different concentrations was also (*E*)-anethole (102.1 mg/g), followed by alpha-himachalene, limonene and Linalool. Structures of the main constituents are reported in Figure 1, quantitative results are reported in Table 1.

As mentioned above, (*E*)-anethole is the major compound present in the EO. Its relative percentages reported in the literature are in the range of 82–90% [24,28,37,38,39,40]. The wide variation in the amount of (*E*)-anethole could be attributed to several factors, namely harvest times [26], extraction technique [41], stages of plant maturity [42], fertilizer type [43], date, collection sites and sowing [23].

In this paper, we included the comparison of the GC-MS analysis of the EO and NA to assess any possible change in the composition of the essential oil during the chemical treatments needed for nanoemulsion synthesis. As shown in Table 1 the qualitative composition is superimposable to the one of the starting essential oil, considering that the amount of oil included in NA is 14% the found percentage of the whole compounds is in agreement with general conservation of the composition, the sum of the amount of the quantified compounds being 122 mg/g. Most of the compounds are in the NA at 8–10 times less, compared to their amount in EO as expected due to introduced EO quantity.

### 2.2. Charachterization of Nanoemulsion

In a previous study, Hashem et al. (2018) evaluated the characteristics of the nanoemulsion by using the same experimental protocol. The *P. anisum* EO-based NE showed Z-average size of 198.9 nm and conductivity of 0.029 mS/cm pointing out the presence of highly conductive ions. Furthermore, the zeta potential was highly negative (−25.4 ± 4.47) which can lead to high degrees of stability [44]. Instead, the PDI value (0.303) indicated a good physical stability of the nanoemulsion, due to the reduced Ostwald ripening [45], with low viscosity of 0.8872 cP, which might be due to the low oil content which delays instability phenomena, resulting in oil droplets with a more homogeneous particle size [46].

### 2.3. Toxicity Assays and Sub-Lethal Toxicity Assays

The results of toxic activity of both the *P. anisum* formulations (EO and NE) against *T. castaneum* are shown in Table 2. Both forms showed significant activity against *T. castaneum* adults in a concentration-dependent manner. At 4 mg/mL concentration, *P. anisum* essential oil (FDI = 76.18%) exhibited the highest feeding deterrent activity and nutritional indices (RGR, RCR, and ECI) compared with *P. anisum* NE (FDI = 1.59%) and control. In addition, the LC_50_ values on adult beetles were 2.1 *v*/*v* (confidence interval 1.8–2.9 *v*/*v*) and 9.8 *v*/*v* (8.6–12.7 *v*/*v*) for EO and NE, respectively (Table 2).

### 2.4. Biochemical Assays

Sub-lethal concentrations (LC_50_) of *P. anisum* EO (2.1 *v*/*v*) and NE (9.8 *v*/*v*) were applied to *T. castaneum* adults to study the abnormalities occurring at enzymatic and biochemical parameters (Table 3). Sub-lethal concentrations (LC_50_) of *P. anisum* EO significantly decreased AST (+30.56%) and glucose (+80.54%). Otherwise, a negative change ranging from 14.75% to 30.56% was recorded in other parameters including total lipid (−14.75%), total protein (−15.81%) and ALT (−17.87%).

On the other hand, treatment with *P. anisum* NE significantly decreased the activity and amounts of most enzymes and macronutrients in *T. castaneum* adults. This decrease was determined as +8.31%, +59.32%, +85.79%, +52.08% and +9.33% in the activities and content of ALT, AST, glucose, total protein and total lipids, respectively (Table 3).

### 2.5. In Silico Molecular Docking Prediction of the Binding Site of Main Compounds of the EO and NE on Key Enzymes

**Figure 2:**
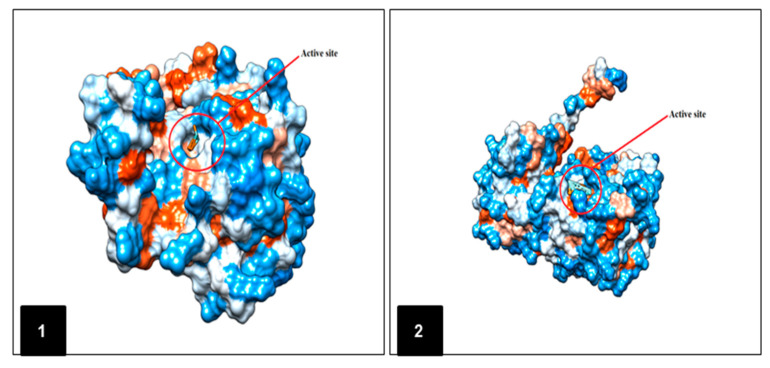
Structure two proteins modeling and their active sites by Chimera molecular graphic software; (**1**) ALT (Alanine aminotransferase) model and (**2**) AST (Aspartate aminotransferase) model.

To the best of our knowledge, this work represents the first study reporting the effect and interaction between an EO-based NE and ALT and AST enzymes of *T. castaneum*. Molecular docking was used to predict the binding site of the major compounds of *P. anisum* EO to AST and ALT enzymes. In the modeler, a least energy model was selected [47]. To obtain the basic data of protein structure in a convenient way, a progression of 3D protein structures was developed by means of homology modeling [48,49]. Basic local Alignment search Tool (BLAST) through Swiss model server was used to build templates, only the 5toq.1. A with AST (Seq. Identity 63.37%) and 3ihj.1. A with ALT (Seq. Identity 59.61%) showed high level of sequence similarity and were selected as templates. The final stable structure of the AST and ALT, and their active sites so obtained are shown in Figure 2.

**Figure 3:**
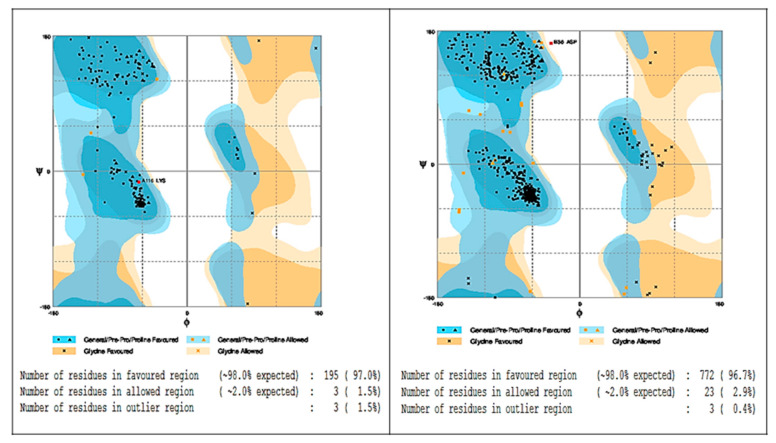
Ramachandran plot analysis: homology models of alanine aminotransferase (ALT, **left**) and aspartate aminotransferase (AST, **right**).

The best protein-based model stabilized, least energy and low RMSD (Root Mean Square Deviation) was obtained with Nano Molecular Dynamics (NAMD) and the graph [50]. The best model was selected on the basis of model evaluation tools, ProSA-web Z-scores and RAMPAGE Ramachandran plots [51] (Figure 3).

**Figure 4:**
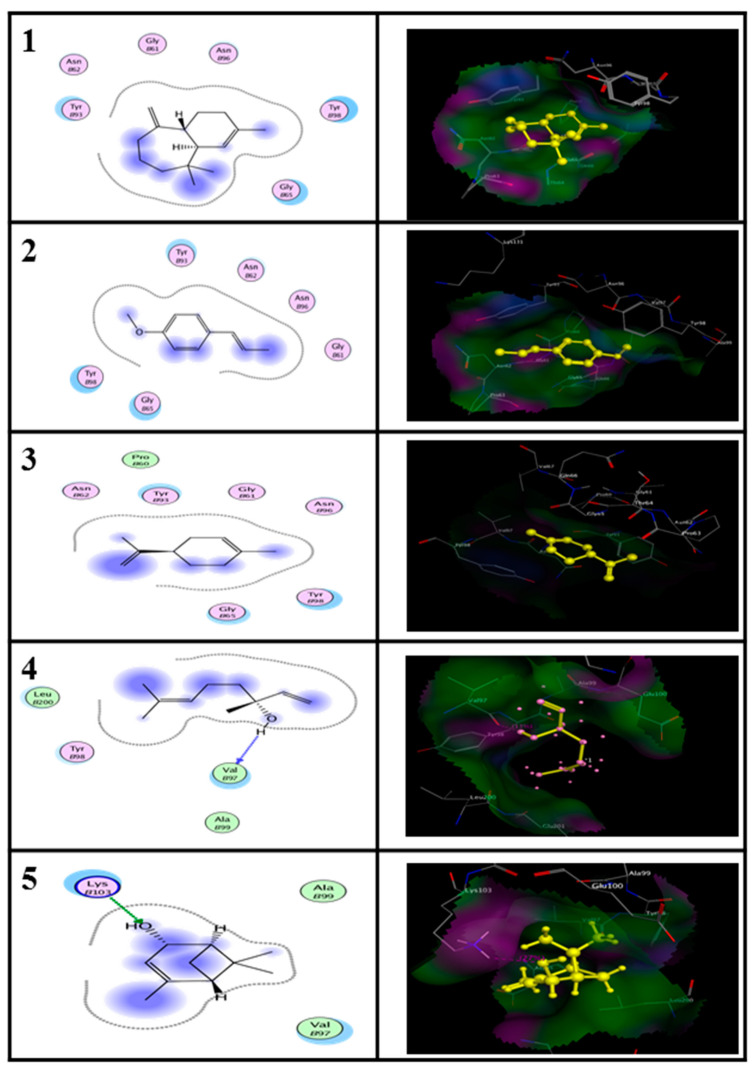
Molecular docking of essential oil (EO) and nanoemulsion (NE) ligands of Pimpinella anisum with homology modeled Alanine aminotransferase (ALT) of Tribolium castaneum created by Molecular Operating Environment (MOE) program.

Based on the GC-MS analysis, the main compounds (≥2%) of EO have been selected as ligands of both proteins modelling). The main constituents were docked into AST and ALT (Figure 4).

**Figure 5:**
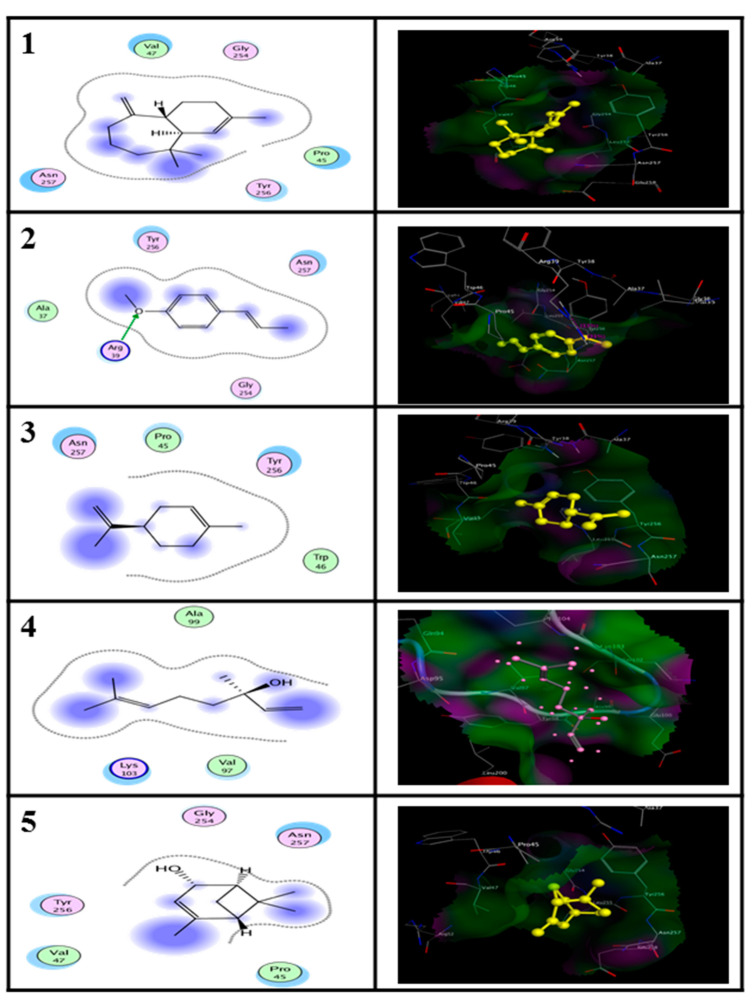
Molecular docking of essential oil (EO) and nanoemulsion (NE) ligands of Pimpinella anisum with homology modeled Aspartate aminotransferase (AST) of Tribolium castaneum created by Molecular Operating Environment (MOE) program.

The information of protein 3D structures is vital for rational drug design. Molecular docking has been performed by Molecular Operating Environment (MOE) software to identify the binding location of the main compounds with both proteins. The 3D structural simulation of the best energy ranked result of the binding mode between enzymes and ligands is shown in Figure 4 and Figure 5. Regarding to the EO form, alpha-himalachalene and (*E*)-anethole compounds showed the most interactions at the lowest energy and strength of the binding of ALT (−12.03 kcal/M) and AST (−11.51 kcal/M), respectively. Regarding to the NE form, alpha-himalachalene and Linalool compounds were the least energy and most closely binding for ALT (−12.03 kcal/M) and AST (−11.95 kcal/M) proteins, respectively.

All results of interaction were observed between four ligands and both proteins (Table 4).

## 3. Discussion

In this work, GC-MS analysis allowed to identify and quantify the main compounds of *P. anisum* EO and to check their amount in the produced NE. The efficacy of *P. anisum* EO and NE as an ecofriendly alternative to synthetic insecticides was then explored significant activity against *T. castaneum* adults was observed in a concentration-dependent manner. Many EOs and their components are known to exhibit antifeedant properties against a wide range of insect pests [4,5]. However, research papers on the effects of aniseed EO against stored grain pests are limited though other Apiaceae species were shown as source of insecticidal agents [9,52,53] and effective against *T. castaneum* [29,54]. The EO from *Azilia eryngioides* (Pau) Hedge and Lamond showed LC_50_ values on *Sitophilus granarius* and *T. castaneum* of 20.05 μL/L and 46.48 μL/L, respectively, after a 24 h of treatment [55]. The EO of *Coriandrum sativum* L. caused a significant decrease of the number of *T. castaneum* larvae reaching the pupal stage and that from pupae to adult stage in a concentration-dependent manner [56].

According to data, there are limited studies of *P. anisum* EO-based formulations against stored pests. Nevertheless, several researchers have evaluated other EOs under nanoemulsions. A previous paper proved that *Pterodon emarginatus* Vogel nanoemulsion works as an anti-acetyl cholinesterase drug against *Aedes aegypti*. Alike, nanoformulation containing 18% of *Lippia sidoides* Cham. EO or thymol killed off 50% of *Sitophilus zeamais* (Motschulsky) adults at concentrations ranging from 1.1 to 3.7 μg/mg [57]. Another work found that *Achillea Arabica* Kotschy, *A. cretica* L. and *A. millefolium* L [19]. EO-based NEs showed fumigant and toxic effects against *T. castaneum*. Previous work found that pulegone encapsulated into coarse NE caused a high mortality (>90%) rates for 5 weeks in *S. oryzae* and *T. castaneum* [58]. Likewise, the eucalyptus EO-based NE containing karanja and jatropha aqueous filtrate, at concentrations of 300 and 1500 ppm, gave 88–100% mortality rates against *T. castaneum* adults within 24 h [59].

A limited number of researchers have, however, paid attention to the effects of *P. anisum* EO and its encapsulated forms on certain enzymes in and outside of the citric acid cycle, glycolytic pathway, and other related biochemical components. In these experiments, therefore, the biochemical effects of *P. anisum* EO and NE on some enzyme of these systems in *T. castaneum* adults were studied.

Considering the main volatile constituents that were measured in the essential oil and nanoemulsion they are well known for their insecticidal activities. As an example previous published papers considered (*E*)-anethole [60]. Anethol resulted very effective as larvicidal and adulticidal agents against *C. quinquefasciatus* [35]. Notably, (*E*)-anethole is able to neutralize the detoxicative system of the insect by interacting with the cytochrome P450 enzyme [61]. Limonene previously studied in this regard and this compound present limited insecticidal properties but low mammalian toxicity [62].

Cedrus atlantica essential oils bearing 14% of beta-hymachalene was, studied for its insecticidal properties against Tribulus confusum [63]; The essential oil of *Artemisia mongolica* containing was studied for repellent and insecticidal properties. Furthermore, in the same study, the major constituents namely Eucalyptol (39.88%), (*S*)-cis verbenol (14.93%), 4-terpineol (7.20%), (−)-camphor (6.02%) and α-terpineol (4.20%) were also evaluated for repellent and insecticidal properties showing significant bioactivity [61]. Thus, the volatile constituents that are present in the EO and NE, overall (*E*)-anethole, can play a crucial role for the bioactivity. For these reasons, we decided to investigate the possible biochemical and molecular targets to understand the possible mode of action of these mixtures.

AST and ALT enzymes are used as indicators of the proper functioning of the fat body in insects and their equivalent in mammals, i.e., the liver [64]. The AST and ALT activities increase with the aging/maturation process in the hemolymph of healthy insects [65]. Exposure to insecticides causes a decrease in the activities of the enzymatic physiological like AST and ALT may impair ATP synthesis, β-oxidation, Krebs cycle, oxidative phosphorylation and other metabolic cycles [66], and may also indicate a decrease in the levels of important dietary proteins to form the amino acids needed to develop tissues, secretions and energy demand [67]. Furthermore, these vital activities in insect tissues may differ from those found in mammals, but in both cases, they can be used to assess the immune status. In addition, the presence of both enzymes (AST and ALT) in insects and mammals (with different sequences between them) and their medical and scientific importance gives us an opportunity to study the side effects of insecticides and their components (whether of the chemical origin or of botanical origin) and know their maximum and minimum damage by molecular docking and The 3D structural simulation. The 3D structural simulation may help us to clarify the mechanism and strength of the binding between proteins and ligands [68].

The results suggest that the analyzed enzymes were found to be sensitive to all treatments with anise EO and its NE. Likewise, previous investigations proved that jasmine and basil EOs significantly increased the activity of AST, while clove EO caused a significant increase of the activity of ALT [69]. In fact, the variable effect of plant EOs on AST and ALT activities might be exerted on the synthesis or functional levels of these enzymes directly or indirectly by altering the cytology of the cells [70]. Furthermore, another work reported that glucose and glycogen provided primary sources for energy under insecticidal stress conditions followed by lipid and cholesterol contents [71]. The glycolytic pathway was probably activated for this purpose. An increase in soluble protein contents was reported in adult beetles of *T. granarium* after 24 and 48 h exposure to phosphine after that they started to decrease [72]. Previous work reported an increase in protein contents in *Rhyzopertha dominica* (F.) after exposure to malathion, a synthetic organophosphorus insecticide [73].

The present investigation demonstrates that the aniseed NE effect is significant compared to the one of EO. This gives the nanoformulation a unique and distinct characteristic, which is the high ability to influence at the molecular levels (cells, proteins, genes, enzymes, etc.), as well as the ability to directly reach the target.

The docking results showed agreement with the ones from enzyme and biochemical assays. This is evident in the compatibility of the biochemical assay with molecular docking analysis (the lowest binding energy with ligands), which showed that the effect on the AST (+60.32) is more affected at exposure to nanoemulsion compared to the ALT (+11.12). In addition, the results confirmed that NE was effective as much as EO against ALT and AST proteins. This leads us to build bio-nanopesticides formulations based on aniseed EO. Notably, these results further substantiate the use of in silico tools for prediction and identifying novel insect repellent compounds. Finally, the current study is considered complementary to the previous study [29] at the applied level, not just the research level, because this study shows the effect of essential oil and its nanoemulsion on insect proteins and enzymes, which are necessary to know the extent of the insect’s ability to show the resistance to these oils in the long term. In addition, the study provides us with knowledge which of the internal components of EO and NE have the ability to bind to insect enzymes, or in other words, which of the internal compounds has the most share in influencing the insect, and then designing biopesticides based on these most influential components.

## 4. Materials and Methods

### 4.1. Insect Rearing

The red flour beetle, *Tribolium castaneum*, was reared on broken wheat grains (whole wheat grains were ground completely in a mortar box then sieved to obtain sizes less than 11 mm) mixed with dried yeast (5%) under laboratory conditions (25 ± 1 °C; 60 ± 3% R.H.), and 10:14 h (L:D) at Stored Products and Grain Pests Department, Plant Protection Research Institute (PPRI), Agriculture Research Center (ARC), Sakha, Kafr El-Sheikh, Egypt. Beetle adults used in the experiments were 7–14 days old. All the following experiments were conducted under similar laboratory conditions.

### 4.2. Essential Oil

Based on our previous researches [29], the aniseed (*P. anisum*) EO was provided by Hashem Brothers Company for Essential Oils and Aromatic Products, Kafr-Elsohby, Kalyoubeya, Egypt.

### 4.3. Nanoemulsion Preparation and Characterization

The NE of aniseed EO was prepared following the method of Hamouda et al. [74] with slight modifications [29,75]. Briefly, coarse emulsion was prepared by mixing aniseed EO (14% *v*/*v*), ethanol (3% *v*/*v*), and biosurfactant non-ionic Tween 80 (3% *v*/*v*), representing 20% (*v*/*v*) of the total emulsion [29]. Then, the coarse emulsion was mixed and kept for 1 h at 86 °C. It was subsequently mixed with water (80%), kept for 3 min at room temperature (25 ± 3 °C) and finally centrifuged at 10,000× *g*. The aniseed NE was stored in dark bottles at ambient temperature until further analysis.

The aniseed NE was characterized by assessing the droplet size distribution (analysis by volume), which was determined by the dynamic laser light-scattering method (DLS). The zeta potential and polydispersity index PDI were investigated by photon correlation spectroscopy using a ZetaPlus tool (Malvern Zetasize Nano-zs90, Malvern Instruments Ltd., Enigma Business Park, Grovewood Road, Malvern, Worcestershire WR14 1XZ, UK) [29].

### 4.4. Gascromatography Coupled with Mass Spectrometry

The GC-MS analysis of *P. anisum* essential oil and nanoemulsion was carried out using gas chromatography-mass spectrometry instrument Agilent 7820A GC 5977B inert MSD single quadrupole (Agilent Technologies, Santa Clara, CA, USA). A HP-INNOVAX column (30 m × 0.250 mm × 0.25 µm) was used. The temperature program was: isothermal at 55 °C and held for 5.5 min. 55–240 °C at 4 °C min^−1^, 4 min hold at 240 °C and 240–250 °C at 10 °C min^−1^ and 5 min hold at 250 °C. The injector temperature was 220 °C. The flow rate carrier gas (helium) was 1.2 mL min^−1^. A spitless injection was used. A total of 3 µL of solution was injected. Samples were prepared taking a volume of 100 µL of essential oil in 1000 µL of ethyl acetate and 50 µL in 1000 µL of tetrahydrofuran:water (8:2) for nanoemulsion, internal standard (nonanol) was added (5 microliters) and solutions were vigorously mixed. Mass spectra were obtained by electron ionization (EI) at 70 eV, using a spectral range of *m*/*z* 45–500. The components were identified by comparison of their retention times and mass spectra with those of WILEY 09 and NIST 12 mass spectral databases, as well as by experimental calculation of the linear retention index, experimentally determined using a mixture of alkanes chromatographed under the above analytical conditions. For quantitative results calibration curves were obtained mixing 100 microliters of nonanol solution (internal standard concentration of 100 µg/mL) with 100 microliters of solutions of reference compounds at four levels of concentrations 200, 100, 50 and 20 µg/mL. Reference compounds were (*E*)-anethole, limonene, linalool, eugenol, cineole, verbenol, *p*-anysaldheyde and trans-cinnamaldehyde. The solutions containing the different ratios of compound/nonanol were analyzed and calibration curves were builted plotting quantity of analyzed compound/quantity of nonanol versus area of analyzed compound/area nonanol. Each reference compound was used to quantify the corresponding compound in the mixture, for the other compounds calibration curves of the most similar standard were used. Obtained values are reported as the average of three different measurements and standard deviation.

### 4.5. Toxicactivity Assay

Determination of toxic activity of the tested EO and NE against *T. castaneum* was performed following the method of Shukla et al. with slight modifications [76]. One mL of the tested EO (diluted in acetone) and the aniseed NE (diluted in deionized water) was mixed in glass flask using a rotary shaker for 15 min with 20 ± 0.0001 g of broken wheat grains (>11 mm) providing the concentrations of 1, 2, 3, and 4 *v*/*v* for the EO and NE, respectively. The treated broken wheat grains were left at r.t. for 15 min allowing the evaporation of the solvent. A control with untreated broken wheat grains (i.e., without oil or nanoemulsion) was maintained under the same conditions.

Twenty adult insects were starved for 24 h, weighed and subsequently placed into each flask. Three replicates of 20 insects were used for each treatment (i.e., EO, NE, and control treatment). After 4 days of the infestation, broken wheat grains weight, weight of live insects and insect mortality were estimated. The nutritional indices were calculated using previous paper [77], as follows: Relative Growth Rate (RGR) = (A − B)/B × No. days, where A = weight of live insects on the fourth day (mg)/number of live insects and B = initial weight of insects (mg)/initial number of insects; Relative Consumption Rate (RCR) = D/B × No. days, where D = biomass ingested (mg)/number of live insects on the fourth day; Efficiency of Conversion of Ingested food (ECI) (%) = (RGR)/(RCR) × 100, the percentage Feeding Deterrence Index (FDI) was calculated: FDI (%) = (C − T)/C × 100, where C = consumption of control broken wheat grains and T = consumption of treated broken wheat grains.

### 4.6. Evaluation of the Potential Mode of Action of Aniseed EO and NE

The lethal concentrations required to kill 50% of the test population (LC_50_) after 4 days for the anise EO and NE were estimated through the concentration-mortality bioassays described above were subsequently used in in vitro biochemical and in silico molecular docking bioassays in order to understand their possible mode of action on adults of the red flour beetle.

### 4.7. In Vitro Biochemical Assays

Sixty adult insects previously exposed to the desired treatments, as described above, were weighed and subsequently homogenize in 2 mL of 0.89% saline solution with the help of motor-driven Teflon glass homogenizer (NIPPI Inc., Tokyo, Japan). The homogenate was centrifuged at 3000× *g* for 30 min in refrigerated centrifuge (Aldo Avenue, Santa Clara, CA 95054 USA) at 4 °C, and the supernatant was separated and used for the in vitro biochemical bioassays using a spectrophotometer [72].

Glucose, total protein and total lipid contents on the beetle extracts were determined by the *O*-toluidine method described by Hafiz et al. [78]. Aspartate aminotransferase (AST), and alanine aminotransferase (ALT) protein activities were determined as previously described [79].

### 4.8. In Silico Molecular Docking Assay

For 3D model building, alanine aminotransferase (ALT) and aspartate aminotransferase (AST) sequences were obtained from National Center for Biotechnology Information (NCBI) server. The sequences of both proteins were submitted by Swiss-Model tools to protein structure homology-modelling more suitable structural template to reliable theoretical for 3D models. Then, these models were analyzed and validated by the Ramachandran’s plot (PROCHECK analysis). Structure models of ALT (Alanine aminotransferase) and AST (Aspartate aminotransferase) proteins and their active sites (pockets) were carried out by Chimera molecular graphic software.

Ligand selection: The main compounds (≥2%) of EO and NE were selected as ligands of both protein modelling. Then, these compounds were obtained from PubChem and Chemspider databases and were prepared by Molecular Operating Environment (MOE) program in MOL format of this ligand and create library of them.

Molecular docking was used to predict the binding site for proteins. The structural simulation helps to clarify the binding mechanism between any proteins and all ligands [68]. Docking steps was performed by using the Molecular Operating Environment (MOE) software package (Chemical Computing Group Inc., Montreal, Canada) as previously described [80]. The proteins and ligand molecules were opened by MOE software. These structures were modified by the addition of hydrogen atoms and energies were minimized using following parameters Force field. The best model obtained from modeler was used for docking analysis. The structure of protein was subjected to 3D protonation and energy minimization using following parameters Force field: MMFF94X + Solvation, Chiral constrain: Current geometry, Gradient: 0.05. This minimized structure was then used as receptors in docking analysis. The active site of protein was found by site finder module of MOE. Docking was run with default parameters of MOE. Once the process was completed, a docked structure indicating the corresponding e-values was generated.

### 4.9. Statistical Analysis

Mortality data were corrected for natural mortality using the Abbott’s formula [81], and the natural mortality did not overcome 20% (ranged between 5−20%). The concentration–mortality bioassays to ascertain the toxicity of EO and NE to the red flour beetle was subjected to Probit analysis [82]. The toxic activity results were subjected to analysis of variance (ANOVA) followed by Tukey’s HSD test (*p* < 0.05). Sigma Plot 12.0 software (Systat Software, Inc. 225 W Washington St., Suite 425, Chicago, IL, USA) was used for all the analyses.

## 5. Conclusions

Our study provided an insight of structure and interactions of ALT and AST protein with essential oils as Nano/Bio-insecticides. The present investigation demonstrates that the aniseed EO-based NE effect is stronger than that of EO. This may suggest that the NE form may give more solubility to mixture in the assay’s conditions and lead to an incremented final effect, or that the NE helps the active constituents to be delivered to the enzyme sites. The observed effects indicate the need for deeper investigations on the possible application of EO-based NEs as pest control agents. Notably, the major constituents of the EO were further filtered using in silico analysis and the binding interactions with the target enzymes were understood by molecular docking studies. Different binding modes between both enzymes and key components may be due to increased insecticidal activity of these compounds and they interacted with more key amino acid residues. Information obtained by the biochemical and theoretical studies was collected to assess the possible role of the active constituents of the EO towards the target enzymes. This information might be interesting in order to develop active ingredients against pests and add new fundamentals about insect defense mechanism based on the other compounds produced by the plants.

## Figures and Tables

**Table 1:** Chemical composition of the essential oil of *Pimpinella anisum* essential oil (EO) and anise nanoemulsion (NE) analyzed by GC/MS.

Retention Time	Compounds	KI *	Compound EO (mg/g)	Compound NE (mg/g)
8.2	Limonene	1.202 (1210)	55.7 ± 0.1	03.2 ± 0.1
8.4	1,8-Cineole	1.207 (1220)	03.6 ± 0.1	0.3 ± 0.1
20.3	Linalool	1.557 (1556)	16.4 ± 0.2	01.9 ± 0.2
20.5	Linalyl formate	1.563 (1579)	09.2 ± 0.1	01.1 ± 0.2
23.8	Methyl chavicol	1.674 (1683)	08.1 ± 0.2	01.1 ± 0.2
24.1	*trans*-Verbenol	1.685 (1683)	24.7 ± 0.2	01.6 ± 0.1
24.2	α-Himachalene	1.688 (1690)	25.2 ± 0.2	03.5 ± 0.1
25.5	Geranial	1.734 (1740)	08.7 ± 0.1	01.2 ± 0.2
28.4	(*E*)-Anethole	1.839 (1847)	801.0 ± 0.2	102.1 ± 0.1
33.1	*p*-anisaldehyde	2.023 (2020)	09.9 ± 0.1	01.3 ± 0.1
33.5	(*E*)-cinnamaldehyde	2.030 (2030)	08.6 ± 0.1	01.2 ± 0.2
36.5	*epi*-α--Cadinol	2.168 (2165)	08.1 ± 0.2	01.0 ± 0.2
36.6	Eugenol	2.170 (2186)	08.9 ± 0.1	01.2 ± 0.1
44.6	Acetyl-isoeugenol	2.475 (2400)	11.3 ± 0.2	01.1 ± 0.1
**Total**			999.4	121.8

* Kovats index were calculated on the basis of the retention time of the analytes compared with a reference mixtures of alkane standard mixtures, tabulated values (NIST) are reported in parenthesis, data are expressed as average of three different measurements and standard deviations.

**Table 2:** Variation of nutritional indices in *Tribolium castaneum* treated with different concentrations of *Pimpinella anisum* essential oil and nanoemulsion during 4 days.

Treatment	Concentration (%, *v*/*v*)	RGR ± SD (mg/mg/day)	RCR ± SD (mg/mg/day)	ECI ± SD (%)	FDI ± SD (%)
*P. anisum* EO	1	−0.25 ± 0.06 b	1.05 ± 0.11b	−17.24 ± 0.84 b	19.04 ± 0.45 c
	2	−0.37 ± 0.28 b	0.79 ± 0.03 b	−34.48 ± 0.21 c	38.09 ± 0.23 b
	3	−0.42 ± 0.05 b	0.52 ± 0.54c	−51.72 ± 0.23 d	57.13 ± 0.04 b
	4	−0.64 ± 0.21 c	0.26 ± 0.21c	−68.96 ± 0.56 e	76.18 ± 0.52 a
	Control	0.13 ± 0.04 a	3.88 ± 0.06 a	34.87 ± 0.21 a	0.00 ± 0.00 d
	LC_50_ (%, *v*/*v*) = 2.1 ^a^ (1.8 − 2.9) ^b^; slope = 2.37				
*P. anisum* NE	1	0.21 ± 0.18 a	3.31 ± 0.46 a	−0.30 ± 1.85 b	0.39 ± 0.21 b
	2	0.14 ± 0.05 b	2.48 ± 0.15 a	−0.61 ± 1.01 b	0.79 ± 0.29 b
	3	0.04 ± 0.06 c	1.65 ± 0.16 b	−0.91 ± 0.32 c	1.19 ± 0.34 a
	4	−0.02 ± 0.02 d	0.83 ± 1.23 c	−1.22 ± 0.34 c	1.59 ± 0.56 a
	Control	0.13 ± 0.04 b	3.88 ± 0.06 a	34.87 ± 0.21 a	0.00 ± 0.00 c
	LC_50_ (%, *v*/*v*) = 9.8 ^a^ (8.6 − 12.7) ^b^; slope = 2.43				

Column means (for each treatment) followed by different letter(s) are significantly different (ANOVA, Tukey’s HSD test, *p* < 0.05). EO = essential oil, NE = nanoemulsion, RGR = relative growth rate, RCR = relative consumption rate, ECI = efficiency of conversion of ingested food, FDI = Feeding Deterrence Index, SD = Standard Deviation, ^a^ Units LC_50_ (%, *v*/*v*) after 94 h.; ^b^ 95% lower and upper confidence limits are shown in parenthesis.

**Table 3:** Effect of 4 day-treatment with LC_50_ of *Pimpinella anisum* essential oil and nanoemulsion on ALT and AST enzymes and major biochemical parameters in *Tribolium castaneum* beetles.

Parameter	Control	*P. anisum* Essential Oil	Variation (%) *	*P. anisum* Nanoemulsion	Variation (%) *
**ALT (U/mL)**	64.8	76.38	−17.87	59.42	+8.31
**AST (U/mL)**	92.5	64.23	+30.56	37.85	+59.32
**Glucose (mg/dL)**	102	19.84	+80.54	14.49	+85.79
**Total protein (mg/dL)**	1.96	2.27	−15.81	0.93	+52.08
**Total lipid (mg %)**	562.5	645	−14.75	510	+9.33

* Variation (%) = [(Control – Treatment)/Control] × 100. ALT = alanine aminotransferase AST = aspartate aminotransferase.

**Table 4:** Molecular docking outcomes of the *Pimpinella anisum* essential oil compounds tested against *Tribolium castaneum*, on the two receptor enzymes, alanine aminotransferase (ALT) and aspartate aminotransferase (AST).

Enzyme	Ligand	Binding Energy (kcal/M)	RMSD (A°)
**Alanine aminotransferase (ALT)**	(*E*)-anethole	−11.93	3.67
	Limonene	−11.26	1.69
	alpha-himachalene	−12.03	2.94
	trans-Verbenol	−10.42	2.42
**Aspartate aminotransferase (AST)**	(*E*)-anethole	−11.51	2.43
	Limonene	−9.34	2.12
	alpha-himachalene	−9.38	3.01
	trans-Verbenol	−7.95	2.74
